# Outcomes of topiramate for prophylaxis of chronic migraine headache

**DOI:** 10.12669/pjms.38.6.5616

**Published:** 2022

**Authors:** Khawar Ahmed, Hussain Rafiq, Shalmeen Tariq

**Affiliations:** 1 Dr. Khawar Ahmed, MBBS, Department of Neurology. Nishtar Medical University & Hospital, Multan, Pakistan; 2 Dr. Hussain Rafiq, MBBS, Department of Neurology. Nishtar Medical University & Hospital, Multan, Pakistan; 3 Dr. Shalmeen Tariq, MBBS, Department of Neurology. Nishtar Medical University & Hospital, Multan, Pakistan

**Keywords:** Topiramate, Migraine, Outcomes. Paresthesia, Prophylaxis

## Abstract

**Objectives:**

To explore the prophylactic outcome and tolerability of topiramate in patients suffering from chronic episodes of migraine.

**Methods:**

A prospective, non-interventional study was conducted at the Neurology Department of Nishtar Medical University & Hospital Multan from 17th Aug 2020 to 17th Aug 2021. The eligible patients were administered topiramate (flexible dose) for six months while the treatment was continued for another six months upon the advice of the physician. The patients were analyzed for improvement in migraine intensity and adverse effects of the evaluated drug at 2^nd^, 4^th^, 8^th^, 12^th^ weeks, and at 6^th^, 9^th^, and 12^th^ months. SPSS version 18 was used for statistical analysis

**Results:**

Out of the total of one hundred enrolled patients, 30 discontinued the study due to unavoidable adverse effects, loss in the follow-up period, and other unknown reasons. The median endpoint dose was 45 mg/dl ± 20.5mg/day. Both median days with migraine episodes and median pain intensity score significantly reduced from 7 to 1.5 days and from 16 to 2.75, respectively (p<0.01). Women who were found to have menstruation-related migraines reported a decrease in the median number of migraine episodes from 3.0 to 0.8 (p=0.01). The utilization of triptan reduced significantly along with significant improvement in self-reported impairment of life. Nausea (2.5%) and paresthesia (5.7%) were the most reported adverse effects.

**Conclusion:**

Topiramate not only significantly prevents migraine intensity and frequency of episodes but is also well-tolerated.

## INTRODUCTION

Migraine is a prevalent neurological disorder that is found to be affecting about 6-8% man and 12-14% of women in the western world.^1^,^2^ Similarly, it has been found that approximately 18% of men and 43% of women experience migraine at some point in their lives.^3^,^4^ Given its strong negative effects on daily productivity and quality of life, its early diagnosis and effective treatment are mandatory.^5^ The effective preventive treatment not only decreases the incidence and severity of attacks but also reduces the migraine-associated socio-economic burden.^6^

Many societies have drafted the criteria that qualify a patient for preventive treatment of migraine 7. In this regard, multiple drugs are in use but none of them is well-supported scientifically in its role to prevent migraine. Generally, recommended migraine preventive drugs include antiepileptic drugs, tricyclic antidepressants, calcium channel blockers, and beta-blockers. 8

Topiramate, a fructopyranose sulfamate, has been proven for its efficiency to prevent migraine in several clinical trials.^9^ Most commonly, the drug of choice is administered as monotherapy; however, in the last decade, growing evidence has also supported add-on therapy.^10^ Although chronic migraine are generally prevalent worldwide but no concrete data is available in Pakistan that specifies the burden of the disease; hence trials on its preventive treatments are scarce. Therefore, we have conducted this trial that not only evaluated the capacity of topiramate in preventing migraine but also assessed its tolerability. This trial will assist both clinicians and the general public in dealing with one of the most under-rated diseases.

## METHODS

A prospective, non-interventional study was conducted from 17^th^ Aug 2020 to 17^th^ Aug 2021 at the Neurology Department of Nishtar Medical University & Hospital Multan. Patients aged older than 18 years and who were diagnosed with chronic migraine according to International Headache Society (IHS) criteria (http://www.i-h-s.org/upload/ct_clas/ihc_II_main_no_print.pdf) whereas patients who had a history of hypersensitive reaction to topiramate were excluded. All were informed of study objectives and their consent obtained. The ethical approval Ref.No#49/114 on Dated 03.08.2020 was sought from the ethical committee of the hospital. All participants were then administered topiramate for six months. However, the dosing period was extended up to 12 months in special cases on the recommendation of clinicians. Similarly, the titration rate was decided on the patient’s response to the therapy. The topiramate therapy was also allowed to couple with other related medications to relieve migraine such as opioids, ergotamine derivatives, triptans, non-steroidal anti-inflammatory drugs, and analgesics if required. Such need for additional medication was recorded along with other data.

All patients were assessed at 2^nd^, 4^th^, 8^th^, and 12^th^ week and later at end of the 6^th^ month. The assessment was also conducted at the 9^th^ and 12^th^ months in patients with prolonged treatment. The primary outcome measures included an investigation of the efficacy of topiramate in preventing migraine and their tolerability. Efficacy was measured by assessing change in the median number of monthly migraine episodes from baseline to the last visit at 6^th^ month or last visit at 12^th^ month (in certain cases). Additionally, variation in both the number of migraine experienced days in a month and pain intensity score from baseline was recorded. The study also assessed the types and incidence of adverse events and the influence of migraine on daily life activity. The following formula was used to measure the daily life impairment score (while keeping 28 days as standard in a month)

**(Days with severe impairment × 3) + (Days with moderate impairment × 2) + (Days with slight impairment × 1)**

Classification of migraine with auras, migraine attacks, and migraine headaches was based on their IHS definitions.

SPSS (version 18.0) was used for statistical analysis. Continuous variables were presented as the interquartile range (IQR), median, and mean along with standard deviation whereas categorical variables were presented as frequency and percentage. Wilcoxon signed-rank tests were used to analyze changes in baseline levels of analyzed variables. Last observation carried forward (LOCF) analyses were carried out for certain variables by using the last available pose-dose value as an endpoint. P-values less than 0.05 were considered statistically significant.

## RESULTS

Out of the total of one hundred enrolled patients, 30 (30%) discontinued the study due to unavoidable adverse effects, loss in the follow-up period, and other unknown reasons. The demographics and disease characters of all hundred enrolled patients are shown in Table-I and Table-II. In 36.4% of enrolled women menstruation-related migraine was found and in 29.5% of patient’s migraine headaches with auras were reported. Only 12 patients (12%) were on active migraine treatment other than topiramate which was continued throughout the study. However, given the scarce data, sub-classification of additional drugs was not done. At baseline 85 patients (85%) were administered a standard dose of 50mg, 7 (7%) had 100 mg, 6 had 200mg and 1 had a baseline dose of 12.5mg/day. After six months of treatments (N=70), 55% patients were having 50mg, 15% were on 25mg dose, 16% on 100mg and 14% were on 75mg per day. The median endpoint dose was 45 mg/dl ± 20.5mg/day.

**Table I:** Demographics of Participants (N=100).

Variables	Data
***Gender (N, %)***	
Female	74 (74%)
Male	26 (26%)
Age, years (mean± SD)	40.5 ± 10.5
Height, cm (mean± SD)	165.7 ± 8.1
Weight, kg (mean± SD)	70.1 ± 12.5
BMI, kg/m2 (mean± SD)	25.5 ± 4.8

**Table II:** Disease characteristic (N=100).

Disease characters	Data
Age when migraine was first diagnosed, years (mean± SD)	26.3 ± 11.0
Time since diagnosis, years (mean± SD)	15.4 ± 9.0
Migraine related to menstruation (female only) (N, %)	
Yes	27 (36.4%)
No	47 (63.5%)
Aura (N, %)	
Yes	29 (29%)
No	71 (71%)

**Fig.1:**
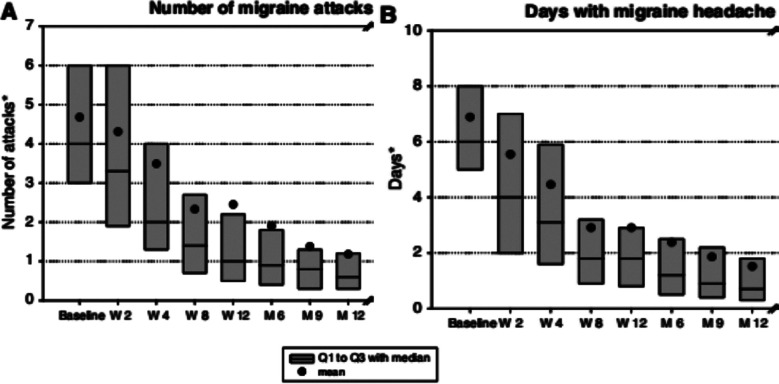
(A) Number of Migraine Attacks (B) Days with Migraine Attack ACROSS Study Period.

Following the 6^th^ month of treatment median number of migraine episodes decreased from 4.5 (2.9-6.5) at baseline to 0.8 (0.2-1.9) in the 6^th^ month (Fig.1A). At-12 month follow up further decline was observed. The reduction in migraine attacks in the overall population was statistically significant. The median number of migraine experienced days decreased from 7.0 (5.5 to 8.5) at baseline to 1 (0.5- 2.75) in the 6^th^ month. The reduction was again statistically significant (p<0.05) (Fig.1B).

**Fig.2:**
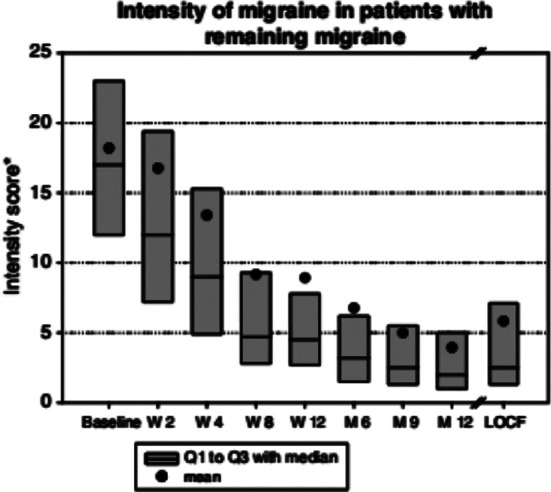
Intensity of pain in patients with remaining migraine.

Significant reduction in pain intensity score across the study period is shown in Fig.2. At baseline 100 patients reported pain intensity of 16 (11-23.5) at baseline to 2.75 (1.0-5.5) at 6^th^ month, reported by 70 patients. In contrast to baseline 29% patients with auras, only five patients of a total of 70 (7.1%) at 6^th^ month and 3 of 50 (6%) at 12^th^ month reported auras.

Efficacy Outcomes related to menstruation-related migraine (MRM) were based on women’s responses. It was found out that women without MRM had a 3.5 (2.75-6.5) median number of monthly migraine attacks at baseline (N=47) which significantly reduced to 0.8 (0.3-2.1) at 6^th^ month (N=30) and 0.7 (0.2-1.3) at 12^th^ month (N=20). In contrast, women with MRM (N=27) had reported median baseline migraine frequency of 3.56 (2.5-6.5) which significantly reduced to 0.8 (0.2-1.5) at the 6^th^ month (N=15) and 0.5 (0.1-1.2) at 12^th^ month (N=10).

**Fig.3:**
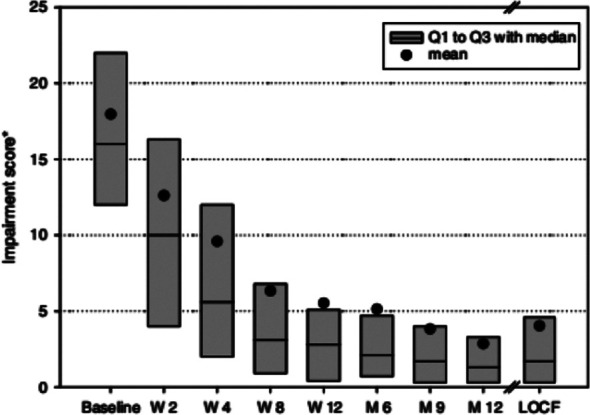
Impairment score of patients.

Daily life impairment in the patients is depicted in Fig.3. The median impairment score reduced from a baseline of 15.5 (10-23.5) points to 2.5 (0.8-4.8) in the 6^th^ month and further to 1.5 (0.2-3.7) points in the 12^th^ month (p=0.001). Likewise, absenteeism reduced from baseline 2.5 ± 2.0 days to 0.5 ± 0.7 at 6^th^ month and 0.1 ± 0.4 days at 12^th^ month (p<0.01).

As a part of acute medication, patients were mainly dependent on triptans in addition to topiramate with a baseline mean intake frequency of 4.5 ± 2.5 days. However, the frequency reduced significantly. At 2^nd^ week, the mean number of triptan treatment days was reduced to 3.7 ± 2.3 days while the frequency stood at 1.5 ± 3.5 days at 6^th^ month and 1.2 ± 2.5 days at 12^th^ month. However, other analgesics were taken for 1.2±2.5 days at 6^th^ month against 7.5 ± 4.3 days on average at baseline and 0.7 ± 1.3 days at 12^th^ month.

Thorough out the study period ten out of seventy 70 patient’s completely followed-up patients experienced adverse events (Table-III). Patients who left the study in the initial weeks of the study due to reports of adverse effects were not included in the final data related to adverse effects since they were assumed to be not related to topiramate intake.

**Table III:** Frequency of patients with Adverse effects (N=70).

Adverse events	Frequency (N, %)
Patients with at least one adverse event	10 (14.2%)
Paresthesia	4 (5.7%)
Dizziness	1 (1.4%)
Diarrhea	1 (1.4%)
Nausea	2 (3%)
Fatigue	1 (1.4%)
Vomiting	1 (1.4%)

## DISCUSSION

The efficacy and tolerability results of this study comply with the previous studies.^9^,^11^ About 14.2% of patients of the study reported at least one adverse effect. Paresthesia was the most reported adverse side effect (5.7%). In the majority of related controlled trials, paresthesia was found to be the most common adverse effect and the frequency varied with a dose of the drug: 35% with 50mg/day; 51% with 100mg, and 49% with 200mg/day, very high when compared with our study. While the incidence of adverse effects was only 6% in the placebo group.^11^,^12^ but the parenthesis incidence has been variable in different studies as in one of the studies where topiramate was administered to epilepsy patients, paresthesia was reported in 8% of patients.^13^ The other major adverse effect in our study was nausea (3%). In contrast, a meta-analysis of randomized control trials reported an 8.9% incidence rate of nausea associated with topiramate treatment. It was also reported in this study that topiramate-associated adverse effects are higher during the titration phase of treatment than the maintenance period. Apart from paresthesia and nausea, dizziness and fatigue were reported to be the most frequent adverse effects in a similar study.^14^ In our study the lower incidence of adverse effects may be associated with a lower mean daily dose of the drug (45mg).

This study was conducted for six months and continued for 12 months in almost 50% of the enrolled patients. The 12-month follow-up showed a further reduction in efficacy-related outcomes. A study reported similar beneficial results of administering the therapy for six months to year.^9^ Similarly, data has suggested that about 50% of patients show improved outcomes after getting migraine preventive treatment for one year.^15^ The efficacy outcomes of our study are supported by another study that found topiramate beneficial in preventing chronic migraine.^16^ The study found that topiramate significantly reduced migraine episodes rates, number of days with migraine, and pain intensity score. In a similar study, Brandes found out that daily intake of 100mg of topiramate reduced migraine frequency up to 40% and up to 42% with 200mg dose.^12^ However, our study found that lower doses of the drug (mean 45mg at endpoint) could be effective in producing significant results.

Menstrual migraine was reported in 36.4% of women. Literature suggests that menstrual migraine are comparatively longer, are more severe, and respond poorly to analgesics. Moreover, pathophysiologically estrogen is associated with menstrual migraine.^17^,^18^ Our study is limited in terms of failure to classify migraine according to IHS standards. However, the treatment proved to be significantly beneficial in preventing such migraine, a result supported by a similar clinical trial.^9^ Lastly, our study also found out improvement in daily life impairment of the affected population, in compliance with already established literature.^19^, ^20^

### Limitation of the study

The study had small sample size, shorter follow-up period, and failure to correlate adverse effects with topiramate since additional medications were also being used.

## CONCLUSION

Topiramate not only significantly prevents migraine intensity and frequency of episodes but is also well-tolerated.

### Authors’ Contribution:

**ST, HR:** Conceived, designed, did statistical analysis & editing of manuscript.

**KA, ST:** Did data collection and manuscript writing.

**KA, ST:** Did review and final approval of manuscript.

**KA, HR:** Responsibile for accountable & integrity of the work.
